# Effects of Age, Gender, BMI, and Anatomical Site on Skin Thickness in Children and Adults with Diabetes

**DOI:** 10.1371/journal.pone.0086637

**Published:** 2014-01-21

**Authors:** José G. B. Derraik, Marius Rademaker, Wayne S. Cutfield, Teresa E. Pinto, Sheryl Tregurtha, Ann Faherty, Jane M. Peart, Paul L. Drury, Paul L. Hofman

**Affiliations:** 1 Liggins Institute, University of Auckland, Auckland, New Zealand; 2 Department of Dermatology, Waikato Hospital, Hamilton, New Zealand; 3 Gravida: National Centre for Growth and Development, Auckland, New Zealand; 4 Auckland Diabetes Centre, Auckland District Health Board, Auckland, New Zealand; 5 Auckland Radiology Group, Auckland, New Zealand; University Hospital Hamburg-Eppendorf, Germany

## Abstract

**Objective:**

We aimed to assess the effects of age, sex, body mass index (BMI), and anatomical site on skin thickness in children and adults with diabetes.

**Methods:**

We studied 103 otherwise healthy children and adolescents with type 1 diabetes aged 5–19 years, and 140 adults with type 1 and type 2 diabetes aged 20–85 years. The thicknesses of both the dermis and subcutis were assessed using ultrasound with a linear array transducer, on abdominal and thigh skin.

**Results:**

There was an age-related thickening of both dermis (p<0.0001) and subcutis (p = 0.013) in children and adolescents. Girls displayed a substantial pubertal increase in subcutis of the thigh (+54%; p = 0.048) and abdomen (+68%; p = 0.009). Adults showed an age-related decrease in dermal (p = 0.021) and subcutis (p = 0.009) thicknesses. Pubertal girls had a thicker subcutis than pubertal boys in both thigh (16.7 vs 7.5 mm; p<0.0001) and abdomen (16.7 vs 8.8 mm; p<0.0001). Men had greater thigh dermal thickness than women (1.89 vs 1.65 mm; p = 0.003), while the subcutis was thicker in women in thigh (21.3 vs 17.9 mm; p = 0.012) and abdomen (17.7 vs 9.8 mm; p<0.0001). In boys, men, and women, both dermis and subcutis were thicker on the abdomen compared to thigh; in girls this was only so for dermal thickness. In both children and adults, the skin (dermis and subcutis) became steadily thicker with increasing BMI (p<0.0001).

**Conclusions:**

Skin thickness is affected by age, pubertal status, gender, BMI, and anatomical site. Such differences may be important when considering appropriate sites for dermal/subcutaneous injections and other transdermal delivery systems.

## Introduction

Skin thickness is affected by a number of factors, including age, gender and body mass index (BMI). Such data may be of importance when determining ideal techniques and sites for intradermal/subcutaneous injections and transdermal delivery systems. This is a particular issue in children with diabetes, among whom subcutaneous insulin injections may be inadvertently delivered to muscle tissue, leading to altered insulin absorption and increased risk of hypoglycaemia [1], [2]. Thus, in this study we assessed the effects of age, sex, BMI, and anatomical site on skin thickness in children and adults with diabetes.

## Methods

### Ethics statement

The study was approved by the Auckland District Health Board Research Review Committee. Written informed consent was obtained from all adult participants. For younger participants, written informed consent was obtained from parents or guardians, as well as verbal or written consent from each child as was appropriate to their age.

### Participants

This cohort was recruited as part of a study evaluating injection techniques in children [3]. Otherwise healthy children and adolescents with type 1 diabetes aged 5–19 years were recruited from the diabetes clinic at Starship Children's Hospital, Auckland, New Zealand. Adults with type 1 and type 2 diabetes aged between 20–85 years were recruited from the Auckland Diabetes Centre, Greenlane Clinical Centre, Auckland. Exclusion criteria included moderate-to-severe lipohypertrophy, other medical conditions such as coeliac disease or autoimmune thyroid disease, associated syndromes (e.g. Down's syndrome), and other secondary causes of diabetes (e.g. cystic fibrosis). Lipohypertrophy was assessed clinically, and, in those subjects who demonstrated mild lipohypertrophy, no measurements were made in areas where any adipose thickening was noted. Participants had weight and height measured, and pubertal status in children were assessed by a pediatric endocrinologist. BMI was calculated for adults, and BMI standard deviation score (BMI SDS) was calculated for each child according to British 1990 standards [4].

**Figure 1:**
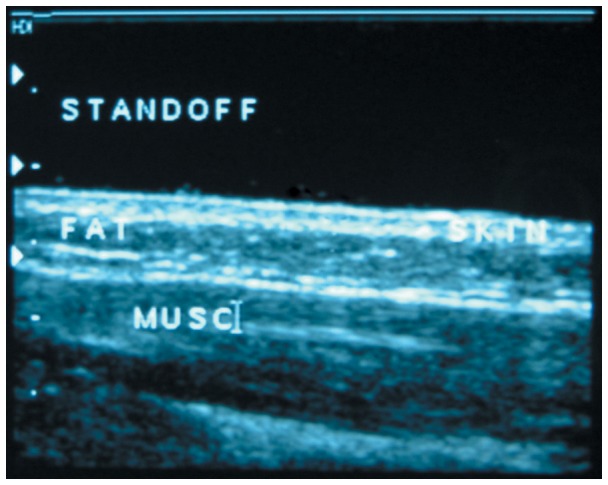
Ultrasound image showing a cross-sectional view of the standoff, dermis (skin), subcutis (fat), and muscle tissue (musc).

Dermis and subcutis thicknesses were assessed using ultrasound at each injection site, in the anterior abdomen 3–4 cm lateral to the umbilicus and at the lateral mid-thigh. Dermal thickness was defined as the distance between the air-skin surface interface and the proximal aspect of the subcutaneous tissue layer, and included the small contribution of the epidermis. Subcutis thickness was measured from the proximal subcutaneous fat boundary to the underlying muscle fascia. Assessments were performed using a Phillips IU-22 ultrasound machine (Phillips Healthcare, Best, Netherlands) and a 17 MHz linear array transducer, having an axial resolution of 0.08 mm [5]. The exact site of needle insertion was marked prior to injection, and the transducer centered over this point. A single measurement was obtained mid-transducer, with cursors centered at the air-skin interface, the skin-subcutaneous fat interface, and the fat-muscle fascia interface. Note that a standoff was used to optimize image quality by increasing the distance between the transducer and the skin (Figure 1). This method of assessing depth of skin layers at injection sites has been well-validated previously [1], [6].

### Statistical analysis

Descriptive data were obtained in Minitab v.16 (Pennsylvania State University, State College, PA, USA). All multivariate analyses were performed in SAS v.9.3 (SAS Institute Inc. Cary, NC, USA). Random effect mixed models with repeated measures were used to assess the association of a number of parameters of interest with dermal and subcutis thickness. Models included age, BMI, anatomical site, and gender as independent variables. For children, BMI SDS rather than BMI was used. Models for children were also run without age, but with the inclusion of pubertal status as a categorical factor. All statistical tests were maintained at a 5% significance level. Age data are presented as means ± standard deviations, while outcome data are presented as model-adjusted means (estimated marginal means adjusted for the confounding factors in the models), with associated 95% confidence intervals.

## Results

### Participants

One hundred and three children and adolescents (54 boys) aged 12.3±3.2 years (range 6.0–19.0 years) were studied. The mean BMI SDS was 0.88±1.08 (range 1.79–3.48). A total of 140 adults (61 men) aged 44.2±14.3 years (range 20.0–81.0 years) were also studied; mean BMI was 28.1±5.8 kg/m^2^ (range: 17.7–45.3 kg/m^2^). A summary of the study's data is provided in Table 1.

**Table 1:** Summary of study results.

		Boys	Girls	Men	Women
**n**		54	49	61	79
**Age (years)**		12.2±3.1	12.3±3.3	43.5±14.3	44.8±19.0
		[6.0–18.0]	[6.0–19.0]	[20.0–72.0]	[20.0–81.0]
**Dermis (mm)**	**Abdomen**	1.89 (1.75–2.03)	1.83 (1.68–1.97)	2.10 (1.99–2.21)	1.99 (1.89–2.09)
		[1.00–5.00]	[1.00–3.40]	[0.80–3.00]	[0.90–3.60]
	**Thigh**	1.60 (1.50–1.70)	1.57 (1.47–1.68)	1.89 (1.78–2.01)	1.65 (1.55–1.76)
		[0.80–2.90]	[0.10–3.00]	[0.90–3.00]	[0.09–3.20]
**Subcutis (mm)**	**Abdomen**	9.13 (7.75–10.51)	13.06 (11.70–14.42)	17.88 (15.93–19.83)	21.26 (19.54–22.99)
		[2.30–26.0]	[2.80–41.0]	[4.0–50.6]	[3.00–58.0]
	**Thigh**	7.68 (6.25–6.12)	13.39 (11.97–14.82)	9.84 (8.21–11.48)	17.68 (16.23–19.12)
		[2.70–18.4]	[4.00–35.8]	[2.30–23.6]	[6.20–60.4]

Age data are means ± standard deviations; all other data are means and 95% confidence intervals (in brackets), adjusted for other confounding factors in the multivariate models. Data ranges are provided in square brackets for each parameter.

### Effects of age, puberty, and gender

Pubertal status had a strong effect on dermal thickness (p<0.0001), which was greater in pubertal than pre-pubertal children. Not surprisingly therefore, increasing age among children and adolescents was associated with a thicker dermis (+52 µm/year; p<0.0001) (Supplementary Figure S1). There was also a positive mild association between age and thickness of subcutis (p = 0.013; Supplementary Figure S1).

**Figure 2:**
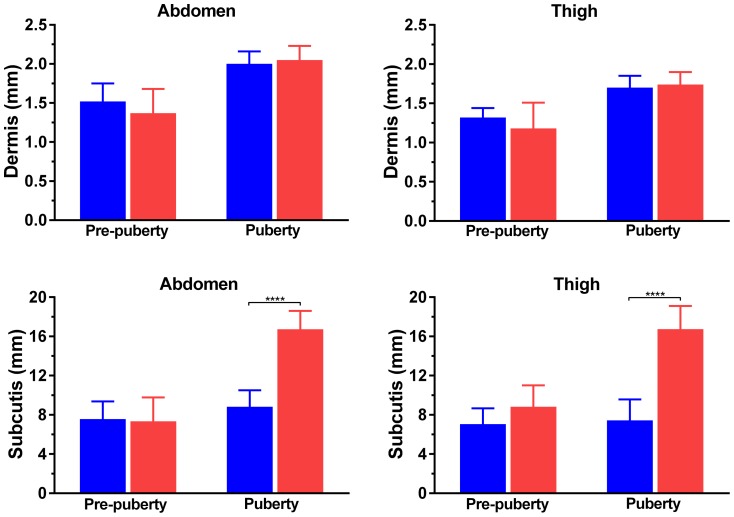
Skin thickness in boys (blue bars) and girls (red bars), according to pubertal status. ****p<0.0001.

Prior to puberty, there were no differences in thickness of either the dermis or subcutis between boys and girls (Figure 2). The dermal thickness in both anatomical sites remained similar among boys and girls in puberty (Figure 2). However, girls displayed a substantial pubertal thickening of the subcutis in thigh (+54%; p = 0.048) and abdomen (+68%; p = 0.009), while there was little change among boys (Figure 2). As a result, the subcutis was considerably thicker in pubertal girls than in boys in both thigh (16.7 vs 7.5 mm; p<0.0001) and abdomen (16.7 vs 8.8 mm; p<0.0001) (Figure 2).

**Figure 3:**
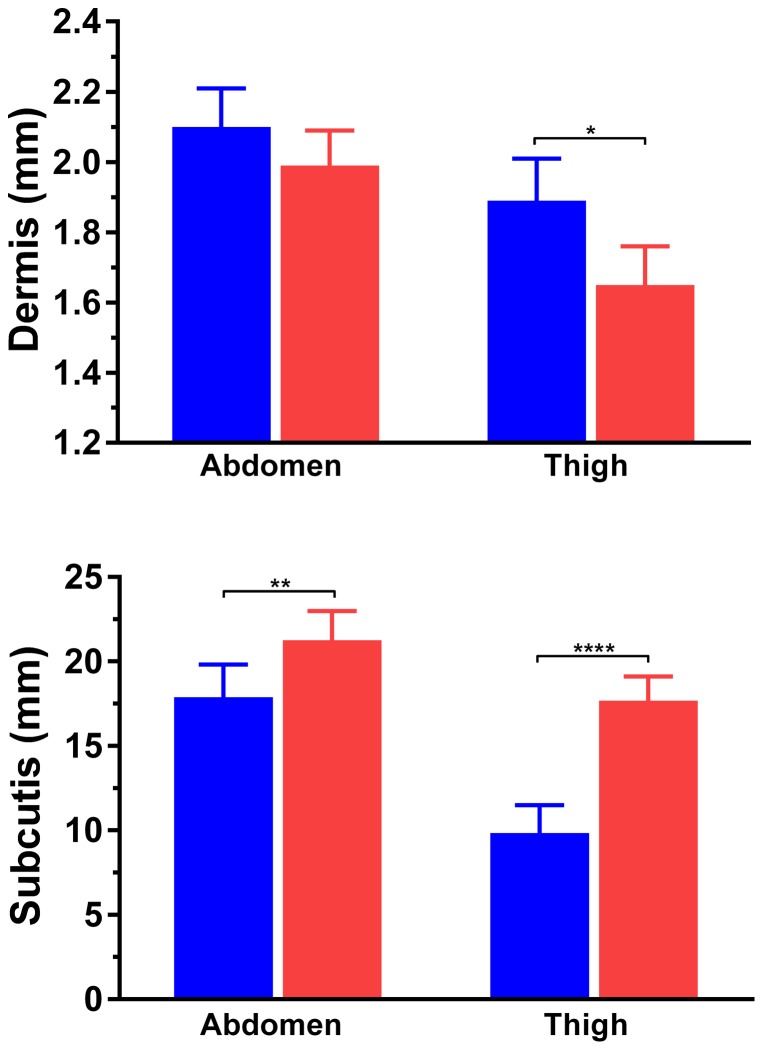
Skin thickness in men (blue bars) and women (red bars). *p<0.05, **p<0.01, and ****p<0.0001.

In contrast to the pattern observed among children, increasing age in adults was associated with a slight decrease in both dermis (−6 µm/year; p = 0.021) and subcutis (−82 µm/year; p = 0.009) thicknesses (Supplementary Figure S1). Abdominal dermal thickness was not different between men and women (2.10 vs 1.99 mm; p = 0.16), but men had a thicker dermis on thighs compared to women (1.89 vs 1.65 mm; p = 0.003) (Figure 3). The subcutis was thicker in women's abdomens than in men's (21.3 vs 17.9 mm; p = 0.012), and even more so on the thigh (17.7 vs 9.8 mm, respectively; p<0.0001) (Figure 3).

### Anatomical site

**Figure 4:**
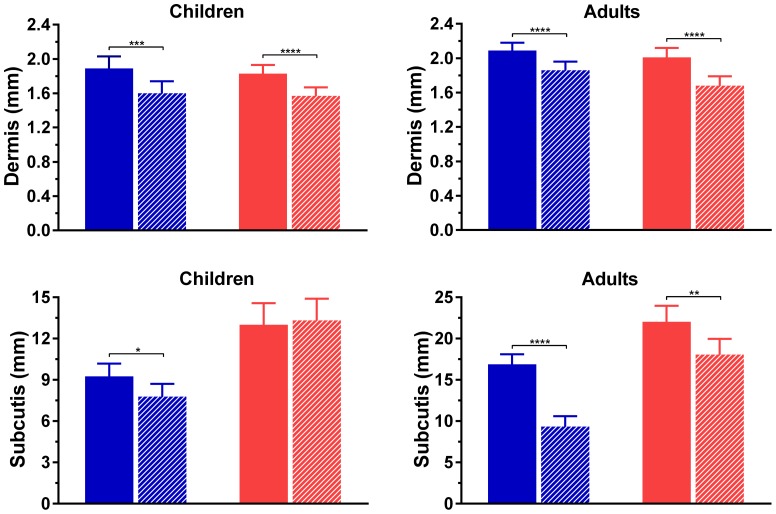
Thickness of skin layers in abdomen (full bars) and thigh (striped bars) in males (blue) and females (red). *p<0.05, **p<0.01, ***p<0.001, and ****p<0.0001 for comparisons between abdominal and thigh measurements.

Boys had abdominal skin that was thicker than their thigh skin (dermis: 1.89 vs 1.60 mm, p = 0.0003; subcutis: 9.25 vs 7.78 mm, p = 0.012) (Figure 4). Girls also had a thicker abdominal dermis compared to their thighs (1.83 vs 1.57 mm; p<0.0001), but there was no difference in subcutis thickness between anatomical sites (13.0 vs 13.3 mm; p = 0.76) (Figure 4).

Among adults, the dermis of men was greater in the abdomen than thigh (2.09 vs 1.86 mm; p<0.0001), with an 80% difference observed in subcutis thickness (16.9 vs 9.3 mm, respectively; p<0.0001) (Figure 4). Women displayed a similar pattern (dermis: 2.01 vs 1.68 mm, respectively; p<0.0001), but had less of a difference in the subcutis (22.0 vs 18.1 mm; p = 0.007) (Figure 4).

### BMI

**Figure 5:**
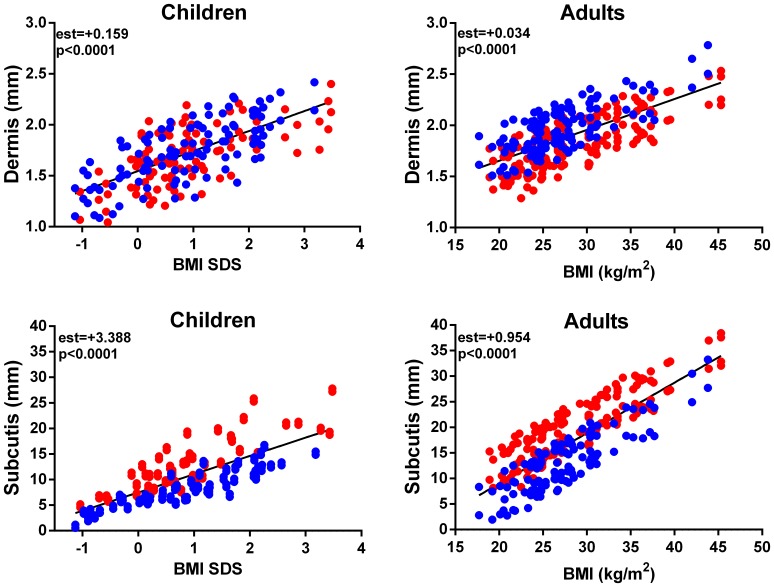
The association of BMI SDS and BMI with thickness of skin layers in children (n = 103) and adults (n = 140), respectively. Data for females are in red and for males in blue.

Skin thickness (both dermal and subcutis) was strongly associated with BMI over the entire life span (p<0.0001; Figure 5). In both children and adults, skin layers became progressively thicker with increasing BMI (Figure 5).

## Discussion

This study on diabetic patients shows that skin thickness is affected by age, pubertal status, gender, BMI, and anatomical site. Similar to our results, Hofman et al. showed no gender differences in the subcutis prior to puberty [2], but pubertal girls had thicker subcutis than boys [1], [2], [7]. Our findings also confirm the existing evidence that adult men have thinner subcutis and thicker dermis than women [8]–[17].

There are relatively limited data on skin thickness throughout childhood, but three other studies have noted an age-related increase in dermis among children [18]–[20]. Considerably more data exist in adulthood, and several studies have shown a thinning of the dermis with increasing age [10], [12], [13], [18], [20]–[24]. In particular, our data are in accordance with the findings of Tan et al. showing a linear increase in dermis until the age of 20, with a subsequent decline thereafter [20]. Although Shuster et al. also found this pattern of decreasing dermal thickness among men, they observed that it was relatively unchanged in women until their 50 s after which it began to decline [25] (probably due to decreasing oestrogen levels after menopause). In regards to the subcutis, at least one other study has observed a decrease associated with ageing [24].

Variations in the thickness of the dermis at different anatomical sites have been shown in numerous studies [7], [8], [10], [13]–[16], [19], [20], [24], [26]. Similar to our findings, one study observed a thicker dermis in abdominal skin compared to thigh skin in adults [8], and a non-significant difference of 0.2 mm in dermal thickness was noted between abdomen and thigh in diabetic children [2].

While an increase in subcutaneous tissue thickness with increasing BMI is obviously expected, other investigations have also found increasing BMI to be associated with a thicker dermis in both children and adults [2], [8], [12], [26]. Conversely, Smalls et al. showed a negative association between BMI and skin thickness on the shoulder of healthy females [27].

Our study shows that skin thickness is affected by age, pubertal status, gender, BMI, and anatomical site in patients with diabetes. This may be of importance when considering appropriate sites for dermal/subcutaneous injections and other transdermal delivery systems, especially given the increasing use of shorter and finer needles. Clearly, skin thickness is an important factor in the selection of needle length for intradermal or subcutaneous injection, particularly for auto-injector devices (e.g. insulin, adrenaline, and biologic response modifiers [28]). Mathematical modeling of transdermal delivery systems however, seldom include the thickness of the dermis and/or subcutis in their models, generally concentrating only on the barrier role of the stratum corneum [29], [30]. For lipophilic drugs, such as testosterone, this may lead to unexpected results [31]. Although this study was conducted in diabetic patients, who have been shown to have slightly thicker skin [32], we believe our data may be extrapolated to the general population.

## Supporting Information

Figure S1 **The association of age with thickness of skin layers in children (n = 103) and adults (n = 140).** Data for females are in red and for males in blue.
(PDF)
