# Supplementary material for: Effects of Age, Gender, BMI, and Anatomical Site on Skin Thickness in Children and Adults with Diabetes
Source: PLoS One. 2014 Jan 21;9(1):e86637. doi: 10.1371/journal.pone.0086637 (PMC3897752; doi:10.1371/journal.pone.0086637)

Figure S1. The association of age with thickness of skin layers in children (n=103) and adults (n=140). Data for females are in red and for males in blue.

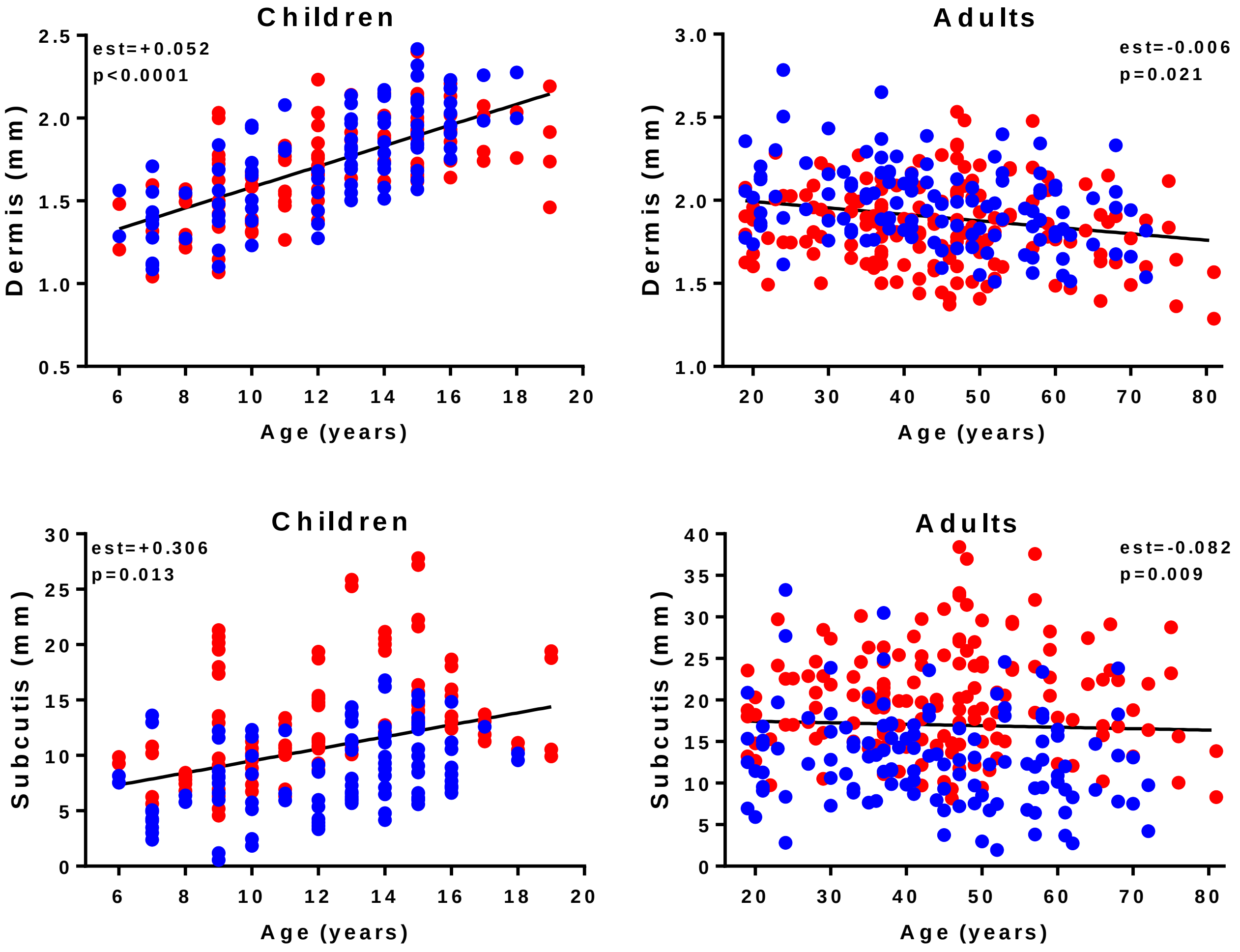

Supplement: Figure S1 — The association of age with thickness of skin layers in children (n = 103) and adults (n = 140). Data for females are in red and for males in blue. (PDF) [file pone.0086637.s001.pdf]
